# Supplementary material for: A comparison of attitudes and knowledge of pre-exposure prophylaxis (PrEP) between hospital and Key Population Led Health Service providers: Lessons for Thailand’s Universal Health Coverage implementation
Source: PLoS One. 2022 May 12;17(5):e0268407. doi: 10.1371/journal.pone.0268407 (PMC9098026; doi:10.1371/journal.pone.0268407)
Supplement: S1 Table — (PDF) [file pone.0268407.s001.pdf]

Table S1 Attitudes towards PrEP service

1.	**Attitude statements in the evidence base**	Strongly disagree	Disagree	Undecided	Agree	Strongly agree
1.1	PrEP is not an effective prevention tool in the “real world”					
1.2	Taking PrEP consistently can prevent HIV infection > 90%					
1.3	Taking PrEP inconsistently would cause ARV resistance					
1.4	Taking PrEP for a long time would lead to more adverse events					
2.	**Attitude statements in service deliverly experiences**	Strongly disagree	Disagree	Undecided	Agree	Strongly agree
2.1	Most patients do not adhere to daily PrEP					
2.2	PrEP will lead to risk compensation (less condom use)					
2.3	PrEP will lead to increased STIs					
2.4	Long-term PrEP use would cause frequent adverse events					
2.5	Patients are likely to be perceived as HIV positive by their partners					
2.6	PrEP would cause patients an increased likelihood of more sexual partners					
2.7	PrEP would result in more needle and syringe sharing					
2.8	Not enough time to engage in PrEP counselling					
3.	**Attitude statements in prioritization**	Strongly disagree	Disagree	Undecided	Agree	Strongly agree
3.1	Behavioral interventions have a greater impact than PrEP on HIV prevention					
3.2	PrEP will have a greater impact than counselling and VCT					
3.3	PrEP should be made available for free to ALL patients who request it					
3.4	PrEP should be made available for free to only those with high risk of acquiring HIV infection					
3.5	Those with no or low risk in acquiring HIV should pay for PrEP if they request it					
3.6	PrEP costs less than care on the HIV epidemic					
3.7	PrEP service should be provided together with condom use counselling and STI testing					
3.8	PrEP should be stopped immediately if patients do not adhere to daily PrEP					
3.9	PrEP should be stopped in patients with frequent STIs					
4	**Attitude statements in effectiveness**	Strongly disagree	Disagree	Undecided	Agree	Strongly agree
4.1	PrEP is effective among MSMs					
4.2	PrEP is effective among TGWs					
4.3	PrEP is effective among serodiscordant couples					
4.4	PrEP is effective among PWIDs					
4.5	PrEP is effective among sex workers					

Support needed for PrEP service

5.	**Support needed from NHSO/MoPH**	Strongly disagree	Disagree	Undecided	Agree	Strongly agree
5.1	PrEP training at least once a year					
5.2	Promotion of PrEP to public through medias and online channels					
5.3	Free PrEP without quota limitation to risk groups					
5.4	Human resource					
5.5	System monitoring and center visit at least once a year					
**6.**	**“PrEP service should be available ……”**	Strongly disagree	Disagree	Undecided	Agree	Strongly agree
6.1	…at all government hospitals under NHSO					
6.2	…at all private hospitals under NHSO					
6.3	…at certified subdistrict health promotion hospitals					
6.4	…at service at qualified CBOs/KPLHS					
6.5	…at certified private pharmacies					
